# Approximation processes in arithmetic in old adulthood

**DOI:** 10.1371/journal.pone.0200136

**Published:** 2018-07-12

**Authors:** Dana Ganor-Stern

**Affiliations:** Psychology Department, Achva Academic College, Achva, Israel; Johns Hopkins University, UNITED STATES

## Abstract

Young and old adults estimated the results of multidigit multiplication problems relative to a reference number. Old adults were slower but slightly more accurate than young adults. They were less affected by the distance between the reference number and the exact answer than the young adults. The same strategies reported by past research–the approximated calculation strategy and the sense of magnitude strategy—were found here. The old adults showed a stronger preference toward the approximated calculation strategy than the young ones, and this probably led to the reduced effect of distance. These patterns are interpreted as reflecting two factors. The first is the extensive experience of the old adults with mental calculation, and the second is the decline in processing speed and in working memory resources with adulthood. The former is responsible for the more frequent use of the approximated calculation strategy and for the higher accuracy of the old adults, while the latter is responsible for their slower responses.

## Introduction

The dramatic increase in the life expectancy during the last decades has facilitated research on the cognitive changes that occur during old adulthood (e.g., [1, 2]). This research has looked at performance in various cognitive tasks, such as arithmetic, memory and decision making. Performance was assessed using quantitative measures of speed and accuracy, but also using qualitative measures of strategy use that might reveal the sources of the differences in speed and accuracy across the ages.

The present research focuses on the effect of aging on numerical abilities. Numerical abilities are important as they are part of the set of skills needed to run a normal everyday life. For example, numerical processes are required for planning the daily schedule or for monitoring the household budget.

Lemaire and Arnauld [3] have shown that when asked to solve multi-digit addition problems exactly old adults were slower than young adults and were less accurate on the difficult problems. Importantly, the number of strategies they used was smaller than the number used by young adults. Touron and Hertzog [4] have trained young and old adults on a small set of complex arithmetic problems. This training is expected to produce a shift from solution using calculation to solution via retrieval. Old adults were more reluctant to switch from calculation to retrieval than young adults, possibly due to their general conservatism and stronger confidence in calculation-based responses.

The present research investigated the effect of aging on computation estimation, which is the ability to solve an arithmetic problem approximately. Computation estimation requires less working memory resources than exact calculation [5] and thus might be especially important in old adulthood, when working memory resources are in decline [1]. One way to test this ability is to present an arithmetic problem (e.g., 37 x 72) and to ask for an approximate answer for it. In such a task individuals use various rounding rules [6–8]. Lemaire, Arnault, and Lecacheur [9] have shown that young adults were faster and more accurate than old adults, especially on the more difficult problems. Individuals across ages used more often rounding down than rounding up strategies. However, they also showed adaptivity in strategy use as they used the rounding down strategy more often when the units digits were smaller than 5 and thus this strategy would introduce relatively little error. Importantly, this pattern of adaptive strategy choice was weaker among old adults than among young ones.

A series of studies that used an inequality-verification task is also relevant for the present research. In these studies participants were presented with addition problems and they had to indicate whether the result is smaller or larger than 100 [10, 11], or than a given reference number [12, 13]. They found split effects, such that performance was enhanced when the given number was numerically far compared to close to the exact answer. They interpreted this effect as reflecting two strategies. Close split problems were assumed to be solved by an exhaustive verification strategy, which involves calculation, while far split problems were assumed to be solved by a non-exhaustive verification strategy, which involves estimation. Duverne and Lemaire [12, 13] found that the split effect was reduced for old adults compared to young adults, and thus concluded that old adults did not use both strategies. Note, however, that none of these studies had an independent measure of strategy use.

## Present study

The present research contributes to the existing knowledge by examining the effect of aging on estimation processes using a different task with another operation, the estimation comparison task with multi-digit multiplication problems. Thus, it tests the generalizability of past findings across tasks and across operations. The use of multi-digit multiplication problems ensures that participants cannot solve such problems exactly without the aid of paper and pencil. Furthermore, the present study includes a direct measure of strategy use based on self reports that was absent in past research [12, 13] .

Specifically, in the current study young and old adults were presented with a series of multi-digit multiplication problems. A reference number was associated with each problem and they were asked to estimate whether the exact answer was larger or smaller than the reference number. The reference numbers were either far or close to the exact answer. After responding the participants reported the strategy they used. Their accuracy, speed, and strategy use were examined to find the extent to which they are affected by age.

This estimation comparison task has been used in a series of studies [14–17]. It has been shown that speed and accuracy of responses were enhanced for smaller problem sizes, for reference numbers that are smaller (vs. larger) than the exact answer and for far (vs. close) reference numbers. Participants reported using mainly two strategies. The first is an approximated calculation strategy, which involves rounding one or two multiplicands, and comparing the product to the reference number. The second is a sense of magnitude strategy, which does not involve any calculation but reflects an intuitive coarse sense of magnitude which is built on the life long experience of solving multiplication problems and on the training provided by the experimental session [17]. The sense of magnitude is faster and requires little working memory resources, but can guarantee a correct response only when the reference number is far. In contrast, the approximated calculation strategy is slower and requires working memory resources, but can guarantee a correct response in all trials. Past research by Ganor-Stern has consistently shown an adaptive strategy choice as both college students and children use the time-consuming approximated calculation strategy more often when the reference number is close to the exact answer (for example, for the problem 27 x 86, when the reference number is 1200). They use the sense of magnitude strategy more often when the reference number is far from the exact answer (for example, for the same problem when the reference number is 500).

Ganor-Stern [15] has looked at the development of performance in this task from childhood to adulthood. With age, there is an improvement in speed and in accuracy. This trend in quantitative measures is accompanied by a shift in strategy use. Fourth graders used mainly the sense of magnitude strategy and with age there was a decrease in the use of the sense of magnitude strategy and an increase in the use of the approximate calculation strategy. This increase might be due to the augmented working memory resources in adulthood and/or to the development of calculation skills.

What will be the developmental pattern in adulthood? This is the main question addressed by the current research. Note that old adults performance in this task might be influenced by at least two factors with conflicting effects. The first is a general decline in speed of processing and working memory resources in old adulthood (e.g., [18, 19]). The second is the advantage for older cohorts in basic arithmetic skills (e.g., [20]).

Old adults are expected to be slower than young adults [3, 9]. Similar to young adults, they are expected to show enhanced performance for trials with reference numbers that are smaller (vs. larger) than the exact answer and that are far (vs. close) from the exact answer, although the latter effect might be reduced [13]. With respect to strategy use, while young adults are expected to favor the approximated calculation strategy [14–17], two predictions might be raised as for the dominant strategy in old adulthood. Since the approximated calculation strategy requires more working memory than the sense of magnitude strategy and since working memory resources decline with age in adulthood (e.g., [18]), the old adults might show a weaker preference for the approximated calculation strategy or they might even favor the sense of magnitude strategy. Alternatively, the long years of practice of old adults in calculation might cause an even stronger preference towards the approximated calculation strategy. Older adults are expected to show weaker adaptivity in strategy choice compared to young adults [9].

To provide another measure of the participants math ability they also performed a short exact calculation task involving multiplication of single digit (D) and 2D numbers. Old adults are expected to be slower than young adults in this task, and this difference might increase for the more difficult problems [3].

## Method

### Participants

Forty-four adults participated in the experiment, with 22 considered old adults, and 22 considered young adults. The age range of the old adults was 65 to 82 years old, with the average age of 72.86. The age range of the young adults was 21 to 31 years old with the average age of 24.77. All young adults were students in an academic institution. Participants' characteristics are summarized in Table 1. The old adults had an average of 15.5 years of education, while the young ones had an average of 13.73 years of education (*T* = 3.40, *p* = .001). The young adults were significantly faster than the old adults (*T* = 4.96, *p* = .0001) in the exact calculation task, but the two groups did not differ in accuracy (p > .15). Participants were paid for their participation 8$ each.

**Table 1:** Participants characteristics.

Variables	Young adults	Old Adults
Number of participants	22	22
Number of females	9	8
Age in years	24.77	72.86
Age range in years	21–31	65–82
Years of education	13.73	15.5
MMSE		27.7
Calculation test–percent error	5.5	9.0
Calculation test–speed (in seconds)	18.8	39.5

MMSE–Mini-Mental State Examination [21]

### Ethics statement

The procedure was approved by the ethics committee of Achva Academic College. All participants provided written informed consent to participate in this study.

### Apparatus

The experiment was conducted on a personal computer with a 17-inch screen. The experiment was programmed using Open Sesame [22].

### Stimuli

To assess the old adults cognitive abilities they were given the Mini-mental test [21] translated to Hebrew. To assess their math abilities they completed a pencil and paper math test composed of five single digit multiplication problems and five 1D x 2D multiplication problems.

The stimuli set for the estimation task was composed of 40 2x2 multiplication problems taken from Ganor-Stern [15]. The exact answers were in the range of 768–8178. Each problem was associated with 4 reference numbers: (1) one which was about one fifth of the exact answer, (2) one which was about one half of the exact answer, (3) one which was about twice the exact answer, and (4) one which was about five times the exact answer. (1) and (4) are the far condition, and (2) and (3) are the close condition. In (1) and (2) the exact answer is larger than the reference number, and in (3) and (4) the exact answer is smaller than the reference number. Reference numbers were rounded to the nearest hundred. The problems were arranged in four lists that were counterbalanced across participants. Thus, each participant responded to only one list. Within each list, each problem appeared once; across lists, each problem appeared with each of the four reference numbers. Within each list, in half of the trials the exact answer was larger than the reference number, and in the other half it was smaller than the reference number. In half of the problems the larger operand was on the right, while in the other half the larger operand was on the left. There were no tie problems in the set of stimuli, and no operand had 0 as units digit.

### Procedure

The experiment was conducted individually in a quiet room. The old adults first filled out a biographical questionnaire, and then completed the Mini-mental test. Participants from both groups performed the exact calculation task on paper and pencil. It included five single digit multiplication problems and five 1D x 2D multiplication problems. Then they completed the computerized estimation task [14]. In each trial, a multiplication problem appeared horizontally on the computer screen with a reference number below it. Participants were asked to estimate whether the answer for each problem was smaller or larger than the reference number. They had to press the "A" key if they estimated it to be smaller, and the "L" key if they estimated it to be larger than the reference number. To make sure that the participants understood the task requirements, they were given two examples, together with the corresponding correct responses. Participants were explicitly told that they should only estimate whether the answer was smaller or larger than the given number, and that they should not solve the problems exactly. The numbers remained on the screen until the participant's response. The order of trials was random. Participants were not allowed to use calculators or paper and pencils for calculation. The experimental set was composed of 40 trials. Participants responded by keypress alone for the first 8 problems, and then for the rest of the 32 problems, they were required after they pressed the computer key for each problem to describe how they reached their answer. The experimenter documented their descriptions.

## Results

The analysis of the estimation comparison task includes analyses of accuracy, speed, and strategy use.

### Accuracy and speed analysis

Responses that took longer than 2.5 standard deviations above each participant mean reaction time (RT) were excluded from the speed analysis (less than 3% of the trials). Percent error (PE) and mean RT for correct responses of each participant were submitted separately to an ANOVA with relative distance between the exact answer and the reference number (far, close), and size of the exact answer relative to the reference number (exact answer is larger than the reference number, exact answer is smaller than the reference number) as within-participants variables, and age (young adults, old adults) as a between-participant variable.

**Fig 1:**
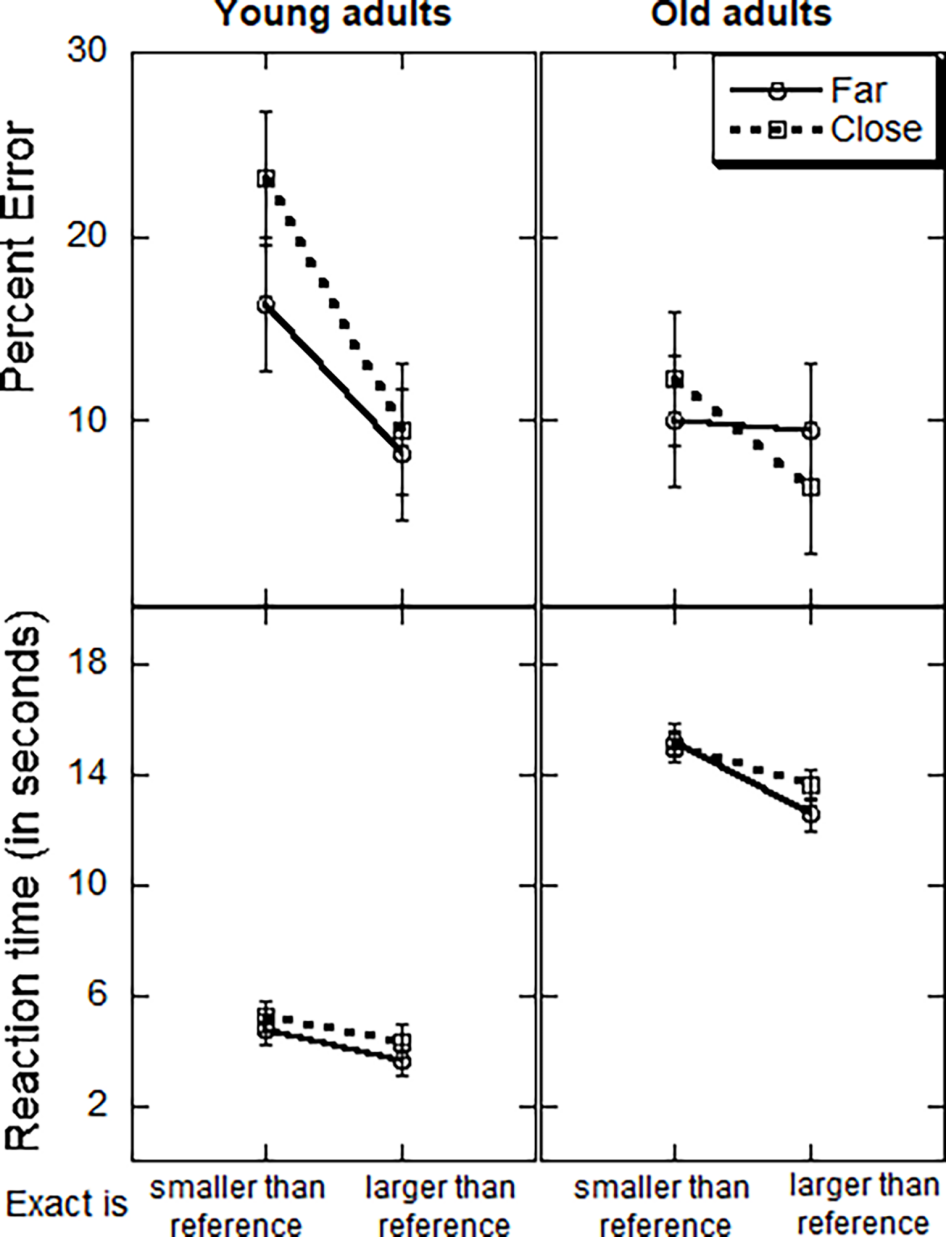
Percentage of errors (top panel) and reaction time for correct responses (bottom panel) by age group, the relation between the magnitude of the exact answer and the reference number, and their relative distance. Bars indicate standard errors computed following Loftus and Mason [23].

As can be seen in Fig 1 (top), the PE of the old adults (9.55%) was lower than that of the young adults (14.32%), although the effect was only marginally significant, *F*_(1, 42)_ = 3.10, *MSE* = 323.59, *p* = .09, η^*2*^_*p*_ = .07. PE was also lower when the reference number was smaller than the exact answer (8.41%) compared to when it was larger than it (15.45%), *F*_(1, 42)_ = 8.90, *MSE* = 145.45, *p* = .004, η^*2*^_*p*_ = .17. As to the expected advantage for far vs. close reference numbers, it was found numerically for the young adults (Fig 1, top), as PE was lower in the far (12.3%) compared to the close condition (16.4%), and not for the old adults, but the interaction effect did not reach significance (*F* = 2.34, *p* = 0.13).

The speed analysis (Fig 1 Bottom) has shown that young adults were faster than old adults, *F*_(1, 44)_ = 21.03, *MSE* = 173.15, *p* = .001, η^*2*^_*p*_ = .32. The average RT was 4.47 sec for the young adults compared to 14.07 sec for the old ones. Moreover, RT was shorter (8.51 sec vs. 10.03 sec) when the reference number was smaller vs. larger than the exact answer, *F*_(1, 42)_ = 7.31, *MSE* = 13.87, *p* = .001, η^*2*^_*p*_ = .15.

### Strategy analysis

Two researchers independently classified the participants self-reports into strategies, based on the experimenter verbatim. The percentage of agreement was 96%. The rare cases of disagreement were settled by discussion. The same strategies that were reported in past research [14–17], the approximated calculation and the sense of magnitude strategies emerged in the present study. As in past research, at the group level the approximated calculation strategy was used more often (80% of the trials) than the sense of magnitude strategy (16%). The preference for this strategy was stronger among the old adults that used it in 87% of the trials, compared to the young adults that used it in 74% of the trials. As in past research, most participants used both strategies (16 old adults and 14 young adults). All the participants that used a single strategy used the approximated calculation strategy.

To examine whether old and young adults differed in their strategy choice, the frequency of strategy use was examined as a function of problem characteristics. This analysis was limited to the 30 participants that used both strategies. An ANOVA was conducted on the number of trials with strategy, relative distance between the exact answer and the reference number, and size of the exact answer relative to the reference number as within-participants variables, and age as a between-participant variable.

**Fig 2:**
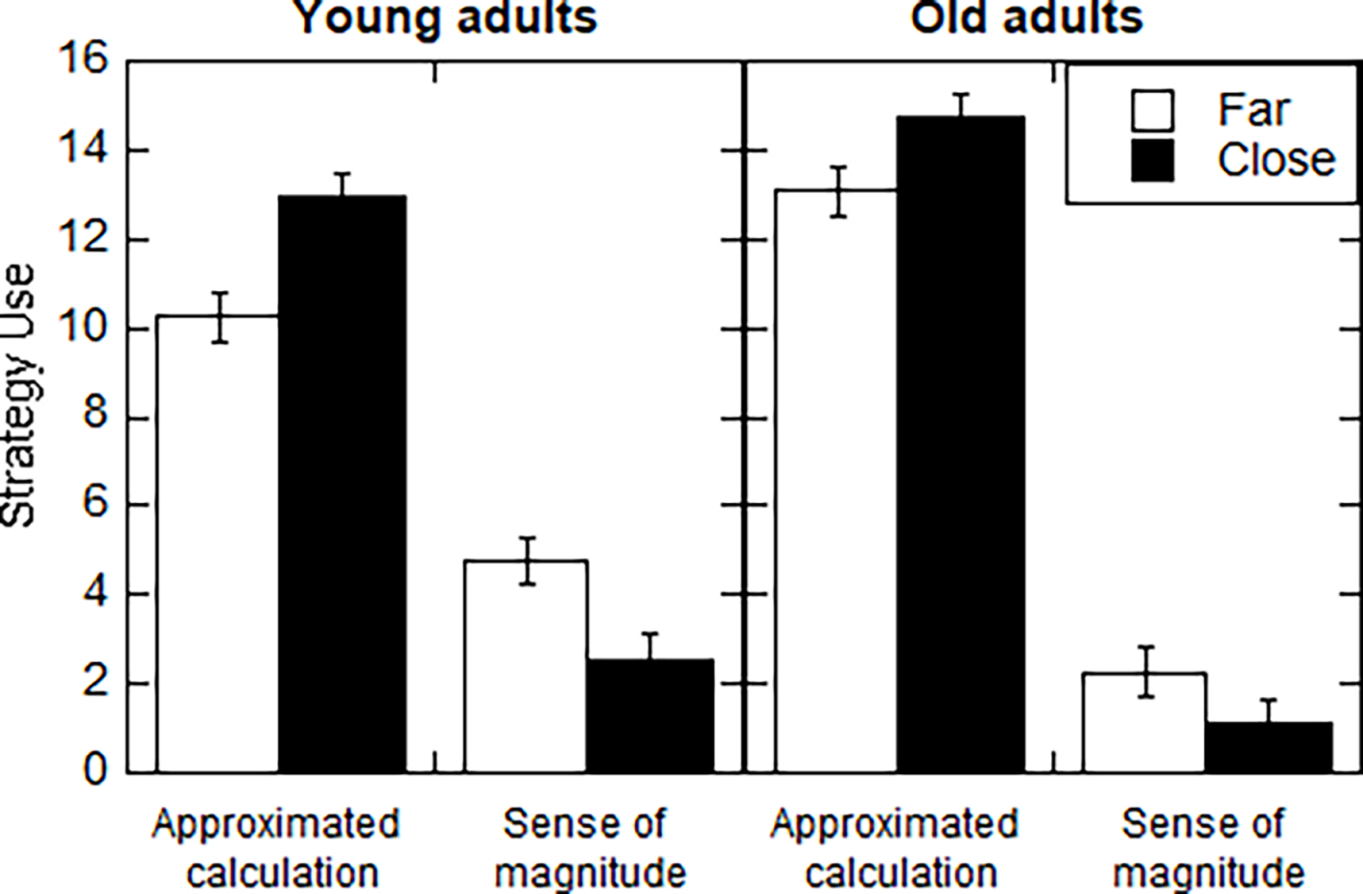
Average number of trials per strategy by age group and distance between the reference number and the exact answer. Bars indicate standard errors computed following Loftus and Mason [23].

The approximated calculation strategy was used more often than the sense of magnitude strategy, *F*_(1, 28)_ = 18.97, *MSE* = 43.89 , *p* = .0001, η^*2*^_*p*_ = .61. As can be seen in Fig 2, the preference for the approximated calculation strategy was stronger among the old adults, *F*_(1, 28)_ = 9.72, *MSE* = 18.39 , *p* = .004, η^*2*^_*p*_ = .18. The interaction between strategy and distance, *F*_(1, 28)_ = 41.41, *MSE* = 2.84 , *p* = .001, η^*2*^_*p*_ = .60 indicated that the approximated calculation strategy was used more often when the reference number was close vs far, *F*_(1, 28)_ = 74.59, *MSE* = 1.54 , *p* = .0001, η^*2*^_*p*_ = .63, and that the sense of magnitude strategy was used more often when the reference number was far vs. close, *F*_(1, 28)_ = 27.31, *MSE* = 1.65 , *p* = .001, η^*2*^_*p*_ = .49. There was also a marginally significant triple interaction between age, strategy and distance, *F*_(1, 28)_ = 3.98, *MSE* = 2.84 , *p* = .06, η^*2*^_*p*_ = .12, indicating that this two-way interaction was reduced for the old adults, suggesting that old adults were less adaptive in their choice of strategies.

### The effect of strategy use on accuracy and speed

**Fig 3:**
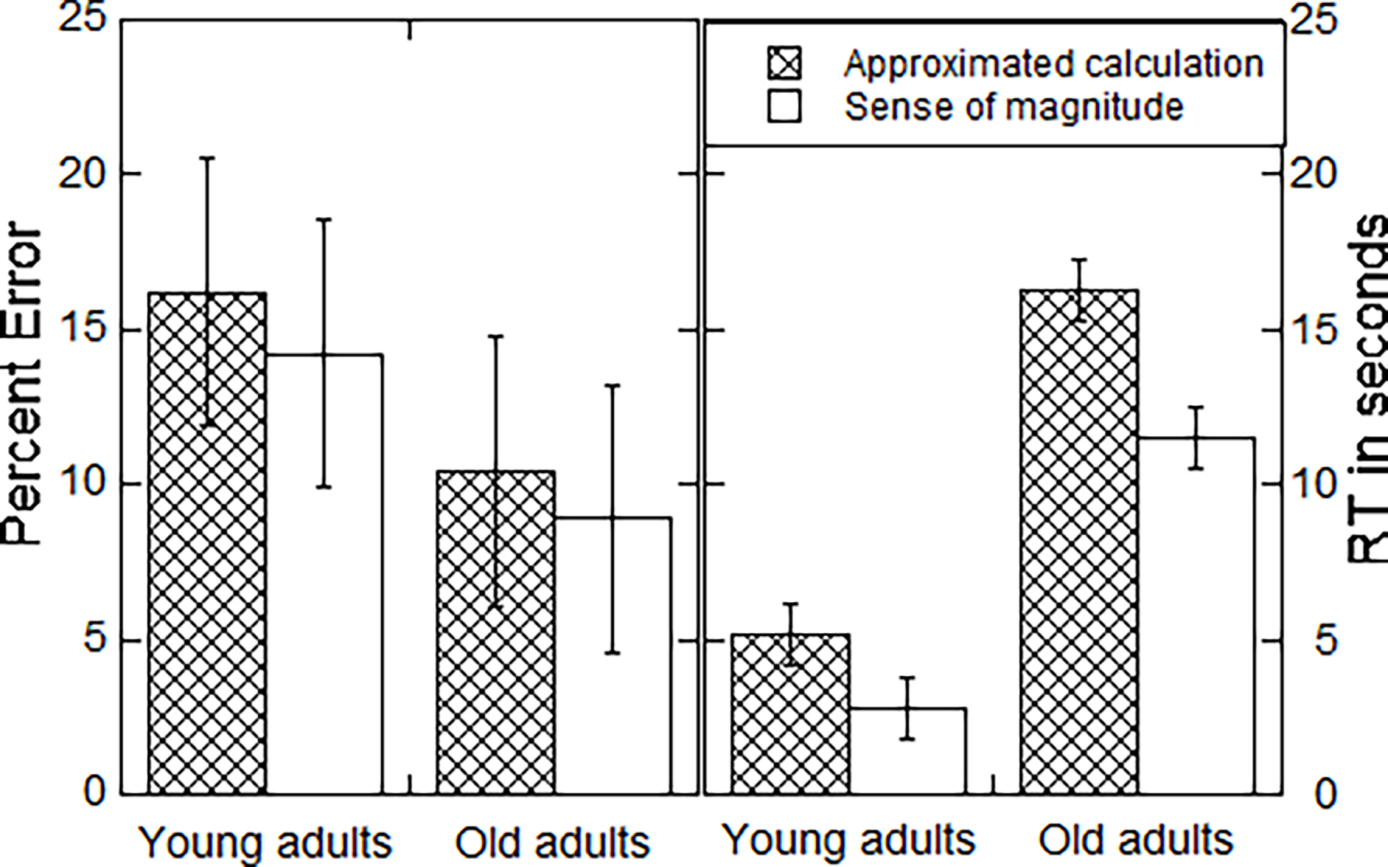
Percentage of errors (left panel) and reaction time for correct responses (right panel) by age group and strategy. Bars indicate standard errors computed following Loftus and Mason [23].

The PE and mean RT of correct responses of participants that used both strategies were submitted separately to an ANOVA with strategy as a within-participant variable and group as a between-participant variable (see Fig 3). The accuracy analysis showed no significant effects. The speed analysis revealed that responses that were based on the approximated calculation strategy (9.77 sec) were slower than those based on the sense of magnitude strategy (7.19 sec), *F*_(1, 23)_ = 10.80, *MSE* = 7.58 , *p* = .004, η^*2*^_*p*_ = .32. The old adults were slower (13.06 sec) than the young adults (3.90 sec), *F*_(1, 23)_ = 16.53, *MSE* = 62.67 , *p* = .001, η^*2*^_*p*_ = .42. The interaction between the two factors was insignificant (*F* < 1).

The previous analysis combines effects of strategy execution and strategy choice. Thus, the longer reaction time of the old adults might be due to the fact that they used the approximated calculation more often than the young adults or it might reflect a general slowness in strategy execution. To examine age differences in strategy execution only we examined the participants that used the approximated calculation strategy in *all* trials (8 young adults and 6 old adults). This analysis has shown the same patterns as the analysis with all participants. Specifically, the old adults were considerably slower than the young adults (11.80 sec vs 5.28 sec for the old and young adults, respectively), *T*_12_ = 2.86, *p* = 0.01. Thus, this analysis suggests that the lower speed of old adults reflect differences in strategy execution. The implications of this finding are discussed in the discussion section.

## Discussion

Old adults were slower than young adults in the exact calculation task and in the estimation comparison task, as predicted based on past research using a variety of tasks [9, 19]. Specifically, in the estimation comparison task, while young adults took on average 4.47 seconds to respond, old adults took 14.1 seconds. This slowness does not reflect a general reduced math ability of the old adults, as their accuracy level was similar to that of young adults in the exact calculation task, and it was even superior in the estimation task (9.5% error for the old adults vs. 14.3 for the young adults).

With respect to strategy use, the two strategies reported in past research—the approximated calculation and the sense of magnitude strategies—were used by the current participants in both age groups [14–17]. Both young and old adults showed a clear preference for the approximated calculation strategy, however this preference was more pronounced among the old adults, consistent with past research [13].

As to the effects of problem characteristics on performance, as predicted, both young and old adults showed the advantage for smaller (vs. larger) reference numbers that was reported in past research [14–17]. Interestingly, the distance effect, which is an advantage for far reference numbers (vs. close ones) that was also reported in past research was not significant in the present study. A closer look (Fig 1) shows that it was present numerically for the young adults in both PE and RT, but not for the old adults.

Duverne and Lemaire [12, 13] found a similar pattern of a decreased effect of distance for old adults in a verification task that requires to judge if an inequality involving an arithmetic problem and a number is correct or not. They have interpreted the split or distance effect in their verification task (i.e., faster responses for incorrect far vs. close trials) as reflecting the use of two strategies, with small split problems responded using a calculation strategy and large split problems responded using an estimation strategy. The decreased split effect among old adults was interpreted as reflecting more frequent use of the calculation strategy and less adaptive strategy choice among old adults. Note however that Duverne and Lemaire [12, 13] had no direct measure of strategy use and they based the conclusion on strategy use on the split effect alone.

The present study both corroborates and extends the results of Duverne and Lemaire [12, 13]. In the current study we provided a direct measure of strategy use based on self reports. Our approximated calculation and sense of magnitude strategies parallel their calculation and estimation strategies, respectively. The current strategy analysis has shown that indeed old adults tended to use the approximated calculation strategy more often than young adults, as suggested by Duverne and Lemaire.

To test Duverne and Lemaire [13] idea that the decrease in the distance effect is due to more frequent use of the approximated calculation strategy, we correlated the number of trials in which the approximated calculation strategy was used with a measure of the distance effect. This measure was calculated as PE or RT for trials with close reference numbers minus PE or RT for trials with far reference numbers. This analysis was conducted on 128 participants, those included in the current study and those of Ganor-Stern (2016). There was a significant negative correlation between the use of the approximated calculation strategy and the distance effect in PE (r = -0.45, *p* < .05) and in RT (r = -21, *p* < .05), thus supporting the link between the reduction in the distance effect and the increased use of the approximated calculation strategy.

The young adults in the present study and in past research [14, 15] tended to use the approximated calculation strategy more often for close reference numbers and the sense of magnitude strategy for the far reference numbers. This pattern was viewed as evidence for an adaptive strategy choice process as it involves using the more time-consuming and attention demanding approximated calculation strategy when it is needed and when the sense of magnitude strategy cannot guarantee a correct response. This pattern was present here, but importantly it was less pronounced for old adults, than for young adults. Evidence for decreased adaptivity in strategy choice among old adults was documented in past research [9, 13]. Old adults tend to repeat the same strategy or even adhere to the same strategy throughout the whole session [9, 24, 25]. This tendency might reflect the weaker executive functions of old adults, which are needed for flexible behavior that involves switching between strategies [26, 27].

Note that the more frequent use of the approximate calculation strategy among old compared to young adults was found despite the fact that this strategy requires more working memory and attentional resources than the sense of magnitude strategy, and that these abilities are in decline in old adulthood [18]. This preference towards using the approximate calculation strategy might be due to their extensive experience with calculation. It might also reflect a tendency towards conservatism and preference for the safer way, even in the cost of speed. This idea is supported by results of Touron and Hertzog [4] who showed that old adults were more reluctant than young adults to switch from calculation to retrieval following practice on a small set of complex arithmetic problems. The safer way in the present task is the approximated calculation strategy which can produce a correct response on all trials, while the sense of magnitude strategy can guarantee a correct response on half of the trials, where the reference number is far from the exact answer.

The old adults were considerably slower than young adults in the estimation task but they were also somewhat more accurate. Can this be a product of the fact that they used the approximated calculation strategy, which usually generates slow but accurate responses, more often that young adults? It seems that this is not the case as the same pattern of longer response times for old adults was found for participants that used the approximated calculation strategy alone.

Thus, the patterns found for old adults might reflect the effects of two factors with opposing influences. The first is the general decline in processing speed and in working memory resources with age [18, 19], which might account for the reduced speed of the old adults in the exact calculation and in the estimation tasks. The second factor is the extensive experience of old adults with calculation, which characterizes their generation, where calculators were not available and mental calculations were more common. There is considerable evidence for cohort differences for basic arithmetic skills, with poorer performance for the more recent cohorts [20, 28]. This might account for the enhanced use of the approximated calculation strategy among the old adults and for their somewhat higher accuracy.

The current results adds to Ganor-Stern [15] that examined performance in this task from childhood to adulthood. Together they show three clear patterns: (1) a continuous increase in accuracy from childhood to old adulthood, (2) an increase in speed from childhood to adulthood, and then a decrease in old adulthood, and (3) a stable increase in the use of the approximated calculation strategy from childhood to old adulthood. Performance in this task might reflect math and calculation skills together with more general characteristics such as speed of processing and working memory. Development from childhood to adulthood occurs for both types of factors [15, 29]. The development of calculation skills and the improved working memory resources presumably lead to the increased use of the approximated calculation strategy with age, and in addition to the enhanced accuracy and speed when executing this strategy. In old adulthood the computational skills seem to stay intact, but there is considerable decrease in speed of processing and in working memory resources [20]. Thus, old adults prefer the approximated calculation and they execute it accurately, but in a much slower pace than young adults.

A cautionary note. First, the current study did not include an independent measure of working memory or executive functions, and thus any explanation of the current results using working memory or executive functions should be taken with caution, and should be addressed directly in future research. Second, the old adults included in the present study had all academic backgrounds, and thus their performance might not reflect that of old adults with lower level of education. Furthermore, as mentioned earlier the performance of the old adults in the current study also reflects the advantage of older cohorts in basic arithmetic skills [28]. Thus, stronger effects of aging might emerge in 20 or 30 years from now, when the children who grew up in the computer era and thus probably used calculators to solve arithmetic problems rather than to solve them by themselves will reach old adulthood.

## Supporting information

S1 Data Percent error and reaction time for correct responses by age, distance, and size of the reference number. (XLSX)
